# Gender sensitivity and its implementation in inpatient cardiology: a mixed-methods study of patient and healthcare professional perspectives

**DOI:** 10.3389/fgwh.2026.1871546

**Published:** 2026-10-16

**Authors:** Sophia Sgraja, Judith Mollenhauer, Martina Kloepfer, Ute Seeland, Clarissa Kurscheid, Volker E. Amelung

**Affiliations:** 1 Institute for Epidemiology, Social Medicine and Health Systems Research, Hannover Medical School, Hanover, Germany; 2 Figus—Research Institute for Health- and System Design, Cologne, Germany; 3 Institute for Gender Health, Berlin, Germany; 4 Department of Internal Medicine/ Gender- and Sex-Specific Medicine, Medical Faculty University Hospital Magdeburg Otto von Guericke, Magdeburg, Germany

**Keywords:** cardiology, clinical guideline, expert standards, gender-sensitive care, healthcare professionals, implementation, mixed-methods, patient perspectives

## Abstract

**Background:**

Gender-sensitive personalized care (GSC+) is a core component of people-centered healthcare and health equity. Evidence from cardiovascular research demonstrates relevant sex- and gender-related differences in symptom presentation, diagnostics, treatment response, and outcomes. These differences are increasingly acknowledged in clinical practice guidelines and nursing expert standards. However, despite growing integration into guideline recommendations, translation into routine clinical practice remains inconsistent. Empirical data integrating both patient and healthcare professional perspectives on guideline-based gender-sensitive care are still limited.

**Methods:**

This exploratory mixed-methods study synthesizes findings from the German health services research project *HeartGap* (funding number: 01VSF22030; DRKS00031317). The study comprised (1) a scoping review on measures, facilitators, and barriers to implementing GSC+, (2) qualitative expert interviews and focus groups with physicians and nurses, and (3) nationwide surveys with physicians (*n* = 155), nurses (*n* = 111), and cardiology inpatients (*n* = 390). Gender sensitivity among healthcare professionals was assessed using the Nijmegen Gender Awareness in Medicine Scale (N-GAMS), alongside measures of perceived implementation of GSC+. Patient perspectives were analyzed descriptively and using multivariate regression models.

**Results:**

Healthcare professionals demonstrated moderate to high gender sensitivity (physicians: *M* = 3.95, SD = 0.73; nurses: *M* = 3.46, SD = 0.76). Perceived implementation of GSC+ was substantially lower, particularly among physicians (*M* = 2.45, SD = 0.59), compared with nurses (*M* = 2.91, SD = 0.81) and patients (*M* = 3.04, SD = 0.65). Higher gender sensitivity did not correspond with higher perceived implementation, indicating a disconnect between attitudes and practice. Although guideline-based knowledge on sex- and gender-related aspects was moderate to high, its translation into routine clinical practice remained limited. Patients evaluated GSC+ positively and reported comparatively low additional needs. Across methods, structural constraints, insufficient operationalization of guideline recommendations, and limited institutional embedding emerged as key barriers.

**Conclusion:**

Organizational, structural, and systemic barriers were consistently identified across study components as relevant factors associated with implementation gaps in GSC+ rather than by negative attitudes or lack of awareness among healthcare professionals. Strengthening the evidence base, operationalizing gender-sensitive recommendations, and embedding GSC+ into organizational routines and education are essential to advance people-centered, equitable, and safe cardiology care.

## Introduction/background

High-quality healthcare requires that diagnostic and treatment pathways adequately reflect the heterogeneity of patient populations. Achieving high standards of care therefore often depends on the ability to stratify patients according to relevant biological, social, and contextual factors. However, across healthcare systems, standardized diagnostic and treatment pathways do not always sufficiently reflect this diversity of patient populations (1). Medical knowledge, clinical guidelines, and routine procedures have historically been developed on the basis of reference groups that do not represent the full range of biological, social, and contextual factors relevant to health (2). This mismatch may translate into unmet needs, inadequate communication, and care experiences that are perceived as insufficiently tailored to individual circumstances (3).

People-centered approaches to healthcare have gained increasing attention. People-centered care emphasizes that individuals should not be regarded merely as carriers of disease, but as persons embedded in social, cultural, and biographical contexts who actively participate in health and care processes (4). High-quality care, from this perspective, is defined not only by clinical effectiveness and safety, but also by responsiveness to patients' values, preferences, lived experiences, and everyday realities. This understanding reflects a broader paradigm shift from a predominantly disease-oriented logic toward a holistic and salutogenic perspective on health and care, placing people and communities rather than institutions or isolated conditions at the center of healthcare systems (5).

Rather than focusing exclusively on technological individualization or risk stratification, personalized care involves aligning diagnostic, therapeutic, and nursing processes with the individual characteristics and contextual circumstances of patients. Within this framework, sex and gender constitute essential dimensions of personalization in care. Biological sex and socially constructed gender roles influence disease risk, symptom presentation, communication patterns, health-related behavior, and responses to medical and nursing interventions. Treating all patients “the same” can therefore maintain inequities when relevant differences are overlooked (1). Gender-sensitive personalized care (GSC+) is thus a requirement and a further step towards truly people-centered and personalized healthcare. At the same time, gender sensitivity is closely interrelated with other dimensions of patient diversity, such as age, socioeconomic position, cultural background, or migration experience, which together shape health needs and care experiences (2). The concept of GSC+ captures this expanded understanding by placing sex and gender at the core of care while acknowledging the intersection with additional dimensions of diversity, including age and life course, socioeconomics, educational level, cultural background, all of which may interact with gender to influence access to care, communication, decision-making, and outcomes (3).

Evidence of sex- and gender-related differences is partially established in cardiology. Evidence has shown that women and men may differ in symptom presentation, especially in acute coronary syndromes. Both sexes most often show chest pain. Women present more often with pain between the shoulder blades, nausea or vomiting, or shortness of breath than men (4). Further symptoms are fatigue/tiredness, pain in the arm, back, neck or jaw, dizziness and indigestion (5). These differences are often assessed against male-oriented diagnostic norms, increasing the risk of delayed recognition and treatment (3).

Gender medicine as a field of research and clinical inquiry first gained momentum in the United States, where early initiatives highlighted the systematic underrepresentation of women in biomedical research and the limitations of a “one-size-fits-all” model of care. In this context, growing evidence began to demonstrate that biological sex and sociocultural gender significantly influence disease manifestation, diagnosis, and treatment outcomes.

One of the key pioneers of this development is Marianne J. Legato, whose work fundamentally challenged the assumption that male physiology could serve as the default model for medical research and clinical care. By systematically demonstrating sex-specific differences in cardiovascular disease, among other conditions, she laid the conceptual foundation for integrating sex and gender into modern medicine (11).

Despite a growing body of evidence and increasing acknowledgment of sex- and gender-related differences in clinical guidelines, standardized cardiovascular care pathways still often remain insufficiently responsive to the needs of heterogeneous patient groups. A discrepancy between available evidence and its translation into routine clinical practice is commonly referred to as gender health gaps. The central challenge is the implementation of existing knowledge in everyday care (6, 7).

GSC+ depends not only on individual professional awareness, but also on organizational structures, interprofessional collaboration, and institutional support. Personalized and people-centered care can only be realized when evidence-based recommendations are embedded in everyday workflows and supported at the system level (8). Practices vary considerably across hospitals, influenced by factors such as leadership commitment, available resources, professional training, and local implementation cultures.

Nursing care plays a key role in the implementation of GSC+. Nurses are continuously involved in symptom assessment, patient observation, communication, and care coordination throughout hospitalization. Their close and sustained interaction with patients positions them as central actors in recognizing gender-related differences in symptom and pain expression, care needs (e. g. cultural/religious particularities of privacy or nutrition), and preferences (e. g. same-sex patient care) (9). From an interdisciplinary perspective, however, GSC+ cannot be understood as the responsibility of a single professional group. Rather, it requires coordinated action across medicine, nursing, and allied health professions, supported by shared standards and institutional commitment.

From the perspective of people-centered care, patients' views are central to assessing whether GSC+ is successfully realized. Patient-reported outcome measures (PROMs) and patient-reported experience measures (PREMs) provide complementary insights into patients' evaluations of their health and care. PROMs capture patients' assessments of health status, symptoms, and functional outcomes, while PREMs focus on experiences of care processes, including communication, information provision, respect, and perceived individualization (10). Empirical evidence on patients' perceptions of GSC+ in hospital settings, however, remains limited and at a general level (11). Moreover, patients and healthcare professionals may differ in their assessments of the relevance of sex- and gender-specific aspects, the amount of information required, and the degree to which care is experienced as individualized.

There is therefore a need for empirical research that examines how GSC+ is currently implemented in everyday hospital practice and that integrates patient perspectives with those of physicians and nurses. Understanding these perspectives is essential for identifying implementation gaps, contextual barriers, and opportunities for change in routine care. Optimizing the healthcare system is essential for high-quality and sustainable care. The quintuple aim, with the factors 1) patient experience, 2) population health, 3) reducing costs, 4) care team well-being, and 5) health equity, provides a framework for the research approach with regard to enhancing healthcare.

This paper provides an overview of the findings of the German health services research project *HeartGap* (Gender health gaps in guideline-based inpatient cardiovascular medical and nursing care and implementation strategies to reduce the gap), funded by the Innovation Fund of the Federal Joint Committee. This article combines a review of previously reported results with the presentation of new data obtained from a patient survey. The aim of the HeartGap project is to systematically assess the implementation status of GSC+ in inpatient cardiology and to develop strategies to reduce persistent gender health gaps (12). This study contributes to a comprehensive understanding of GSC+ as a core component of people-centered healthcare.

The following Figure 1 shows the architecture of the mixed-methods study. A key contribution of this article is the integration of findings from previously published studies to synthesize the different components of the HeartGap project and derive recommendations for action.

**Figure 1:**
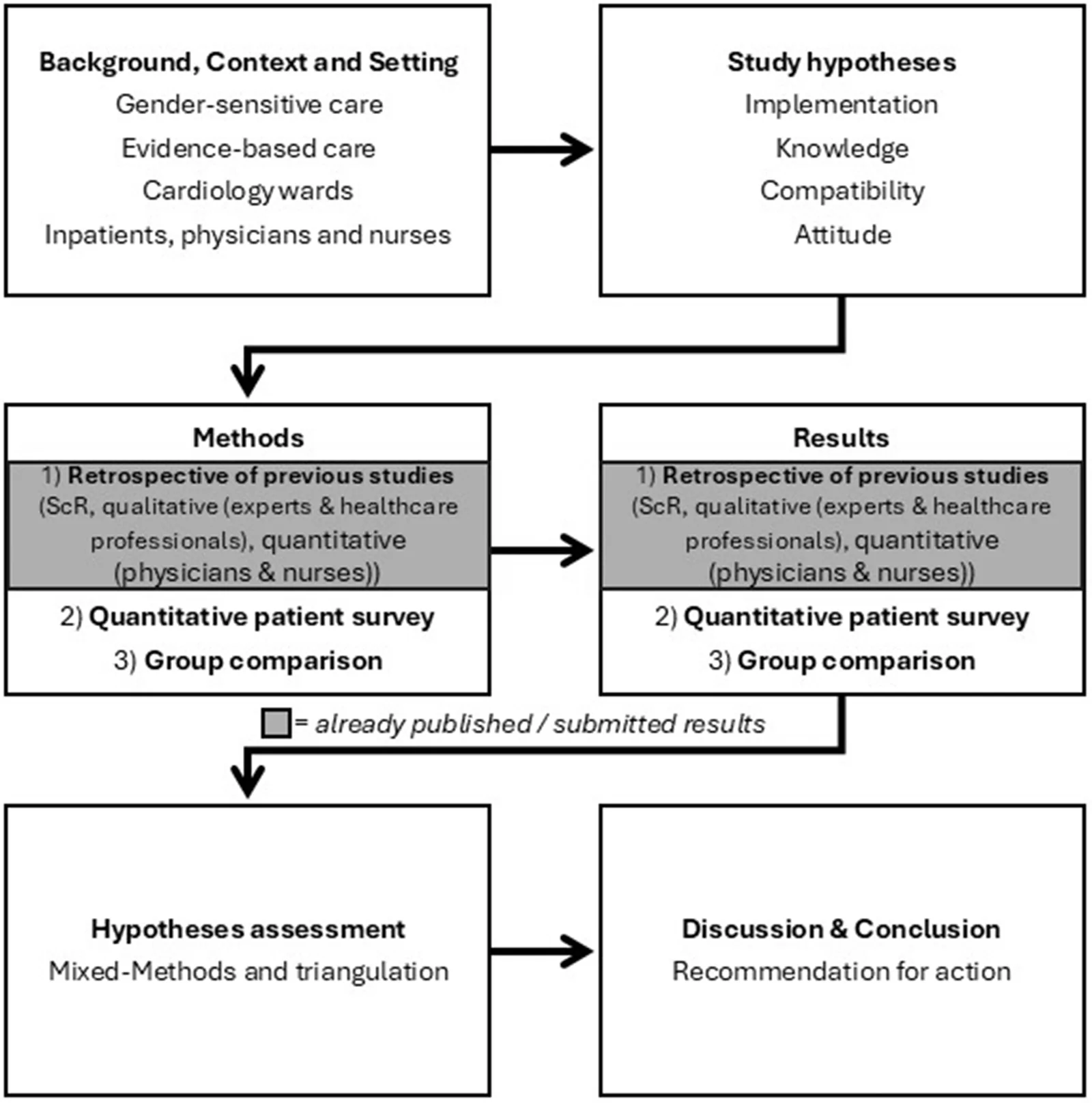
Architecture of the article.

## Context and setting

Between 01.01.2023 and 30.06.2025 in the health research project HeartGap funded by the Innovation Fund of the Federal Joint Committee (funding number: 01VSF22030) the implementation status of gender-sensitive personalized care and influencing factors is investigated. This study is registered in the German Clinical Trials Register (DRKS) under the clinical trial registry number DRKS00031317 (registration date: 24 February 2023. For recruiting adequate samples, hospitals with cardiology departments or internal medicine units focusing on cardiology were identified through the German hospital register. To ensure representativeness of considered hospitals and participants, the characteristics “level of care” (hospital with ≤500 beds, hospitals with 501-800 beds, hospital >800 beds, teaching/research hospital with >800 beds) and “regional location” (rural community and city with <100,000, and large city with ≥100,000 inhabitants) are considered (12).

## Study hypotheses

The summarizing mixed-methods study explores the perspectives of different stakeholder groups (patients, physicians, nurses, and experts) regarding GSC+. The aim is to examine similarities and differences in perceptions of GSC+ and to identify potential needs for action in healthcare practice. Based on the underlying research objectives, explicit hypotheses were derived and are assessed at the end of the article following presentation of the results. Hypotheses 1–5 were already described in the study protocol (12):

1. Despite the availability of gender-sensitive recommendations in clinical guidelines and expert standards, their implementation in routine clinical practice remains limited, partly due to insufficient knowledge among healthcare professionals.
2. Working conditions, particularly staff shortages and economic constraints, are key factors contributing to the low level of implementation of GSC+.
3. The requirements for gender-specific differentiation as formulated in clinical guidelines and expert standards are insufficiently known among healthcare professionals.
4. The requirements for gender-specific differentiation as formulated in clinical guidelines and expert standards are not fully compatible with everyday clinical practice.
5. The requirements for gender-specific differentiation as formulated in clinical guidelines and expert standards do not fully correspond to the attitudes of healthcare professionals.

## Methods

The sequential mixed-methods study is divided into three main parts: 1) scoping review, 2) qualitative study with experts and healthcare professionals (physicians and nurses), and 3) quantitative study (healthcare professionals and patients). This article focuses on the evaluation and presentation of the results of the patient survey, framed by the previous published parts of the study by stakeholders and literature (12). The following Table 1 provides an overview of the study components and participation in the study presented:

**Table 1:** Overview of study parts.

Study parts	Components	Sample	Instrument	Publication status	Contribution to the present status
Scoping review	Literature overview	117 included articles	Systematic literature search	Published	Consideration to derive recommended actions
Qualitative study part (physicians)	Focus group interviews	41 participants, 9 groups	Semi-structured interviews	Under review	Consideration to derive recommended actions
Qualitative study part (nurses)	Focus group interviews	39, participants, 9 groups	Semi-structured interviews	Published	Consideration to derive recommended actions
Quantitative study part (physicians)	Questionnaire survey	155 participants	Questionnaire	Published	Group comparison, recommended actions
Quantitative study part (nurses)	Questionnaire survey	111 participants	Questionnaire	Published	Group comparison, recommended actions
Quantitative study part (patients)	Questionnaire survey	390 participants	Questionnaire	Present study	Novel data and group comparison
Group comparisons	Questionnaire surveys	133 Physicians, 111 nurses, 390 patients	Questionnaire	Present study	Novel results, recommended actions

The present study follows six Good Reporting of A Mixed Methods Study (GRAMMS) reporting criteria (13) (Supplementary Appendix 1).

### Review of previous studies

First, the scoping review aimed to identify measures, facilitators, and barriers to enhance a sustainable implementation process of GSC+ based on international and national (German) literature. A systematic literature search was conducted (searching period: 06/07/2023 to 15/11/2023), and the literature was identified from the databases PubMed, Web of Science, and CINAHL following the Preferred Reporting Items for Systematic reviews and Meta-Analyses extension for Scoping Review (PRISMA ScR) (14). Several search terms with keywords like “women”, “men”, “sex”, “gender medicine”, “implementation”, “measures”, and “gender sensitivity” were built with Boolean operators. The search results cover fifteen years (2008–2023) and are composed in English or German. In addition, snowball principle, hand search, and grey literature screening were conducted. The relevance of 23,508 articles was assessed, from which 117 articles were included in content analysis. The included articles were subsumed in one of the four derived main dimensions (i) politics, (ii) research/science, (iii) education, and (iv) institutions of care (15).

Second, expert interviews with stakeholders and focus group interviews with healthcare professionals, divided into physicians and nurses from hospitals' cardiology departments in Germany, were conducted following the criteria for reporting qualitative research (COREQ) checklist (16).

The expertś interview aim (*n* = 8; female = 4, male = 4) was to identify implementation measures from different perspectives. Between February 2023 and July 2024, guided interviews were conducted with experts, audio taped, and transcribed. The interview guidelines were divided into three sections: 1) introduction (perceived development of GSC+ and relevance/acceptance), 2) implementation, and 3) research needs. The content analysis using MAXQDA resulted in 7 main categories and 16 subcategories.

The healthcare professionals' interviews (*n*_physicians_ = 41, *n*_nurses_ = 46) aimed to identify the definition of gender medicine and gender-sensitive nursing care, influencing factors (facilitators/barriers), and implementation measures for GSC+. Between June 2023 and May 2024, guided interviews were conducted with experts, audio taped, and transcribed. The interview guidelines were divided into three sections: 1) definition of gender medicine/ gender-sensitive nursing care (9), 2) influencing factors (e.g., perceived development of GSC+ and relevance/acceptance), and 3) implementation measures. The content analysis using MAXQDA software was oriented on the methodology of qualitative content analysis according to category formation according to Kuckartz (17).

Third, the quantitative nationwide questionnaire survey was conducted online from May 2024 to February 2025 with physicians (*n* = 155) and nurses (*n* = 111) (18, 19). The questionnaire is divided into three parts: 1) questions about the participants, 2) gender-sensitivity assessed using the validated Nijmegen Gender Awareness in Medicine Scale (N-GAMS) (20–22), and 3) knowledge about gender-sensitive content in medical guidelines respectively degree of implementation of gender-sensitive content from nursing expert standards. The collected data was evaluated descriptively and using inferential statistics.

Finally, the results of the mixed-methods study were consolidated into recommended actions and were discussed with an expert advisory council (*n* = 6).

### The quantitative patient survey

During the empirical data collection, patients were also asked in a nationwide questionnaire survey about their perspectives and needs on GSC+ in hospitals on cardiology wards. The research questions of the patient survey are:

1. How do patients evaluate GSC+ in German hospitals, and what are their needs within this context?
2. To what extent do patients report awareness of sex-related cardiovascular differences, and where do they obtain information on these topics?
3. Is the data collected related to the characteristics of the patient or the hospital?

### Inclusion and recruitment

The study was conducted between May 2024 and February 2025. The target group was inpatients from cardiology wards and internal medicine with cardiology departments.

The hospitals were chosen from eight strata of selected attributes (number of inhabitants and degree of care). The strata are divided into two categories according to population size (large cities with more than 100,000 inhabitants, cities with up to 100,000 inhabitants) and the hospitals are divided into four categories according to level of care, defined as the number of hospital beds (teaching and research hospital with > 800 beds, large hospital with > 800 beds, small to medium-sized hospital ≤ 800 beds). Some of the patient surveys were conducted at the hospitals where the focus groups for caregivers took place. Additional hospitals were recruited through an open invitation process via e-mail.

The patient surveys were conducted in 14 hospitals, partly iteratively, resulting in 27 survey appointments in the six federal states of North Rhine-Westphalia, Lower Saxony, Bavaria, Saxony, Saxony-Anhalt, and Mecklenburg-Western Pomerania in Germany. Researchers of the HeartGap project accompanied the paper-and-pencil survey with patients.

Table 2 shows the distribution of participants per strata. In total, 390 patients were included in the study with the following inclusion and exclusion criteria:

Patients 1) with chronic and acute heart disease, 2) ≥18 years old, 3) insured in statutory health insurance, and 4) with no cognitive impairments.

**Table 2:** Distribution of participants per strata.

Strata no.	*n*
1	75
2	63
3	67
4	93
5	36
6	56

Strata no.1 = teaching/research hospital in large city, strata no. 2 = teaching/research hospital in city, strata no. 3 = hospital with > 800 beds in large city, strata no., strata no. 4 = hospital with 501-800 beds in large city, strata no. 5 = hospital with ≤ 500 beds in large city, strata no. 6 = hospital with ≤ 500 beds in city.

### Patientś questionnaire

Patient participation was conducted on an anonymous and voluntary basis. Prior to enrollment, all participants provided informed consent. The study received a positive ethics vote from Hannover Medical School.

The questionnaire is divided into three parts with a total of 17 questions. The first part includes questions about socio-demographics such as sex, age, migration background, graduation, and professional qualification. The questionnaire included a single item on participants' sex/gender (“Geschlecht”). Sex assigned at birth and current gender identity were not assessed separately, and no response category for gender-diverse identities was included. In the second part, self-developed questions are based on the contents of the chosen medical guidelines (Medical guidelines: German Cardiac Society (DGK) guidelines: Therapy of acute myocardial infarction in patients with ST-segment elevation (STEMI) (20), Non–ST-segment elevation acute coronary syndrome (NSTE-ACS) (21), Guidelines of the Association of the Scientific Medical Societies in Germany (AWMF): S3 National Disease Management Guideline for Chronic Coronary Heart Disease (22), European Society of Cardiology (ESC) guidelines: ESC Guidelines for Acute Coronary Syndromes in patients without persistent ST-segment elevation (2020) (20), ESC Guidelines for the Management of Acute Coronary Syndromes (2023) (23) and German nursing expert standards: Decubitus prophylaxis (24), patient discharge management (25), pain management (26), fall prevention (27), continence promotion (28), care of chronic wounds (29), nutrition management (30), patients with dementia (31), oral health (32), skin integrity (33), and promotion of mobility (34). These questions ask for individual menu selections, patient-physician communication, information and interaction, and patient-nurse interaction. The answer options are coded with 1-4, whereas the third item of the six-item index is reverse-coded.

The third part includes two items assessing patients' self-reported awareness of sex-related cardiovascular differences, specifically potential differences between women and men in myocardial infarction symptoms and blood pressure. These items were intended as an exploratory assessment of whether sex-related cardiovascular differences were familiar to patients rather than as an objective test of cardiovascular knowledge. Furthermore, there is a question about which media the respondents used to obtain information on gender-sensitive care. The final question was: To what extent do you think gender-sensitive care is implemented in the hospital ward? Optional comments on the topic could be made. The original and translated questionnaires are added to the Supplementary Additional File.

### Data preparation

The data were downloaded from the online questionnaire platform, LimeSurvey, and checked for plausibility by a researcher. In preparation for the study, an *a priori* calculation was conducted to determine the distribution across strata (12). The data set concerning the strata distribution was then examined, and absolute frequencies were recorded. Given the larger sample size compared to the *a priori* calculation (*n* = 388) and the deviating distribution, the data were weighted to minimize potential distortions in the results. To achieve this, the *a priori* calculation and absolute frequencies of the survey were divided by the total sample (*a priori* data *n* = 388; survey *n* = 390). The weighting factor for each stratum was then calculated by dividing the relative frequency from the *a priori* calculation by the relative frequency observed in the survey data. The weights per stratum are shown in Table 3. The survey data set was weighted for descriptive statistics (23).

**Table 3:** Weights per strata.

Strata no.	*A priori* calculation (%)	Survey	Weights
1	7.22	19.23	0.38
2	2.06	16.15	0.13
3	3.09	17.18	0.18
4	13.14	23.85	0.55
5	19.07	9.23	2.07
6	55.41	14.36	3.86

Strata no.1 = teaching/research hospital in large city, strata no. 2 = teaching/research hospital in city, strata no. 3 = hospital with > 800 beds in large city, strata no., strata no. 4 = hospital with 501-800 beds in large city, strata no. 5 = hospital with ≤ 500 beds in large city, strata no. 6 = hospital with ≤ 500 beds in city.

A total of 390 patients participated in the questionnaire survey conducted between May 2024 and February 2025 (*a priori* sample size calculation: *n* = 388). The questionnaire was structured into three thematic sections: Demographic information (sex, age, country of birth, educational and professional background), hospital stay, and GSC+ (person-centered meal selection, communication with physicians, same-gender care, and implementation of GSC+), self-reported awareness items (gender-specific blood pressure and heart attack symptoms, media representation). The questionnaire comprised 17 items in total.

#### Survey procedure

The patient surveys were conducted across 14 hospitals, partly in an iterative process, resulting in 27 survey sessions in six federal states: North Rhine-Westphalia, Lower Saxony, Bavaria, Saxony, Saxony-Anhalt, and Mecklenburg-Western Pomerania. One to two research staff members were present at each survey session to invite patients to participate and, if necessary, assist with understanding the questions. The questions and response options were read aloud, and answers were recorded accordingly on the questionnaire; patients also had the option to complete the questionnaire independently on their own.

#### Inclusion and exclusion criteria

- Chronic and acute heart conditions
- Age ≥ 18 years
- Statutory health insurance coverage
- No cognitive impairments

#### Analysis strategy

The data analysis focused on patients' opinions and perceptions regarding GSC+ and theirself-reported awareness of sex-related cardiovascular differences. For descriptive analysis, absolute and relative frequencies were calculated. The influence of personal characteristics such as age, the recorded sex/gender variable, country of birth, and educational background on both knowledge and the perceived need for GSC+ (each measured as a mean index) was examined using multivariate linear regression models. For the following analyses, the recorded binary sex/gender variable was used to compare women and men without attributing observed differences specifically to either sex or gender. Dummies were created for school and educational attainment, whereby, due to the small number of cases, patients with and without other school qualifications were combined with secondary school graduates, and patients with doctorates and university degrees were combined into one category. People in training or studying and with other vocational qualifications were declared as missing values, as the categories cannot be meaningfully assigned to another.

### Statistical methods

The entire analysis was performed using the statistics software IBM SPSS version 29. Initially, the absolute and relative frequencies were calculated for each question. Subsequently, multiple linear regression was used to examine the relationship between the variables of sex, age, migration background, level of care, region, and the patients' needs/perceptions, as well as patientś knowledge regarding GSC+. In preparation for the multivariate analyses and due to the exploratory character of the research, a backward elimination of variables was conducted in the preliminary analysis with all patient-related independent variables.

### Additional cross-sample and mixed-methods analysis (patients, physicians, nurses)

Mean comparisons between the samples of patients, nurses, and physicians were performed to determine group differences. The analysis aimed to obtain a holistic overview of the status quo in German hospitals. The consolidation of all results from the mixed methods analysis and the involvement of a panel of experts led to the derivation of participatory recommendations for action. The experts were selected via purposeful sampling and invited to a workshop. The results of the mixed-methods study were previously provided to them, and they were discussed and adjusted during the workshop. The experts (*n* = 6) included a cardiologist, a nursing scientist, a professor of public health, an equal opportunities commissioner, a scientist, and a health insurance executive.

## Results

The results section begins with a summary of findings from the scoping review (15), the qualitative focus groups (9), and the quantitative surveys with physicians (19) and nurses (18). The main analytical focus, however, is placed on the evaluation of the quantitative patient survey.

### Results of scoping review

The scoping review aimed to identify measures, facilitators, and barriers for the sustainable implementation of GSC+ in healthcare practice. The identified content was assigned to four overarching implementation dimensions: Politics, research and science, education, and institutions of care; Reflecting interdependent levels that jointly shape implementation processes.

**Within the political dimension,** legislative frameworks, targeted funding programs, and policy-driven strategies were identified as key mechanisms for establishing GSC+ sustainably. Political commitment increases the visibility of GSC+ and creates binding structural conditions, for example through the legal anchoring of gender considerations and gender-sensitive research funding requirements. In contrast, missing binding regulations, insufficient political prioritization, and political instability were described as major barriers to long-term implementation.

**The research and science dimension** highlights the importance of systematic evidence generation. The integration of sex- and gender-related aspects in study design, data collection, analysis, and reporting was identified as essential for building a robust evidence base. Scientific institutions and funding bodies can support this process through mandatory gender criteria, training initiatives, and gender-sensitive publication standards. Barriers include inadequate consideration of gender in research planning, underrepresentation of women and gender-diverse populations, and limited translation of evidence into clinical guidelines.

**The education dimension** emphasizes the role of qualifying healthcare professionals to deliver GSC+. Mandatory integration of GSC+ into curricula, teaching materials, and assessments across all levels of education was identified as a key facilitating factor. Effective implementation further depends on sensitized educators, institutional support, and sufficient time and resources. Limited curricular space and lack of trained faculty are commonly reported obstacles.

**The dimension of institutions of care** focuses on organizational implementation measures, including quality circles, diversity or gender officers, and adapted documentation systems. Leadership commitment, organizational culture, and interprofessional collaboration were found to influence implementation success. However, limited resources, time pressure, staff shortages, and insufficient institutional accountability continue to hinder consistent implementation.

Overall, the scoping review demonstrates that implementing GSC+ is a complex, interdependent process requiring coordinated action across the four dimensions. Sustainable implementation depends on an overarching strategy that links political frameworks, scientific evidence, education, and organizational structures rather than relying solely on individual commitment.

### Results of the qualitative focus groups with physicians and nurses

The qualitative focus groups with physicians and nursing professionals from cardiology departments provide complementary insights into the current understanding and implementation of GSC+ in inpatient practice. Across both professional groups, GSC+ was perceived as relevant, yet its integration into routine clinical processes was described as fragmented and largely unsystematic.

From the physicians' perspective, discussions revealed a biomedical-focused understanding of GSC+. Most participants associated GSC+ primarily with sex-specific differences in disease prevalence, symptom presentation, and pharmacological response. Cardiological examples, particularly unspecific symptom presentation in women with acute coronary syndrome, were frequently named. Alongside this dominant biomedical framing, some physicians also referred to social and communicative dimensions of gender, including interaction patterns, unconscious biases, or gendered expectations in clinical encounters. These aspects, however, were mentioned less consistently and were not systematically integrated into clinical reasoning. Overall, implementation of GSC+ was described as inconsistent and strongly dependent on individual initiative rather than institutional structures.

Physicians identified multiple barriers to implementation, most notably time constraints, high workload, and a lack of structural anchoring within clinical guidelines, education, and hospital routines. Although clinical guidelines were described as the primary reference for decision-making, gender-specific recommendations were perceived as scarce, inconsistently presented, or insufficiently operationalized for daily practice. As a result, GSC+ was often regarded as an additional rather than integral component of standardized care.

Nursing professionals described practice-oriented and relational understanding of GSC+. Their definitions extended beyond biomedical aspects to include communication, protection of privacy, same-sex care during intimate procedures, and sensitivity to patients' emotional and psychosocial needs. Nurses also emphasized the relevance of additional diversity factors such as age, cultural background, religion, language, and physical or mental condition, highlighting the close link between GSC+ and broader concepts of patient-centered care. Similar to physicians, nurses reported that GSC+ was rarely formalized in institutional standards or standard operating procedures and was instead implemented intuitively based on individual experience (9).

Structural barriers were also prominent in the nursing focus groups. Participants emphasized time pressure, staff shortages, and organizational constraints as major barriers to consistently providing GSC+. While gender- and diversity-related needs were frequently recognized in patient interactions, nurses reported limited opportunities to address these needs systematically within existing workflows. Management support, targeted training, and organizational prioritization were identified as important but largely missing enablers.

Across both professional groups, the findings reveal a discrepancy between awareness of gender-related differences and their structural implementation in inpatient cardiology care. GSC+ was largely practiced in an informal and situational manner rather than being embedded in institutional processes, education, or quality management systems.

### Results of quantitative survey with nurses and physicians

The quantitative surveys among physicians and nurses working in inpatient cardiology departments provide complementary insights into gender sensitivity, profession-specific knowledge, and the perceived implementation of GSC+ in everyday clinical practice.

Measured with the Nijmegen Gender Awareness in Medicine Scale (N-GAMS), physicians showed a high level of gender sensitivity (*M* = 3.95, SD = 0.73), indicating a strong awareness of the clinical relevance of sex- and gender-related differences. In addition to attitudinal measures, physicians' knowledge of sex- and gender-specific aspects of cardiovascular care was assessed using guideline-derived items. The mean knowledge index was 0.63 (SD = 0.08), reflecting moderate to high overall knowledge. Nevertheless, item-level analyses revealed relevant gaps, particularly in relation to sex-specific diagnostic thresholds and pharmacological differences. These findings suggest that, despite a generally solid knowledge base, specific and operationalizable knowledge remains incomplete (19).

In contrast to their high gender sensitivity and moderate to high knowledge levels, physicians perceived the implementation of GSC+ in routine clinical practice as limited. The perceived degree of implementation was rated as moderate to low (*M* = 2.45, SD = 0.59). Multivariate analyses showed that working in a university hospital was positively associated with higher knowledge levels. No significant associations were found between gender sensitivity, knowledge, and the perceived degree of implementation, indicating that higher individual awareness or knowledge did not correspond to higher perceived implementation.

Among nurses, gender-sensitivity (N-GAMS) was at a moderate to high level overall (*M* = 3.46, SD = 0.75). Nurses showed high agreement with items emphasizing biological differences between women and men and the general importance of gender sensitivity for care quality. Lower agreement was observed for items related to the relevance of gender in non–sex-specific conditions, uniform treatment of women and men, and the role of gender in nurse–patient communication, pointing to heterogeneity in how gender-sensitive principles are integrated into everyday nursing practice.

With regard to institutional realization, nurses perceived the implementation of gender-sensitive nursing care (GSNC+) as comparatively high. On average, nurses rated approximately 70% of GSNC+ as implemented in their hospitals (*M* = 0.70, SD = 0.15). Substantial variation emerged across organizational contexts. Nurses working in university hospitals and in hospitals with more than 800 beds reported a significantly lower degree of implementation than those working in smaller hospitals. Regression analyses further indicated that nurses born in Germany reported higher gender sensitivity than nurses born in other countries (18).

### Results of quantitative patient survey

A total of 390 patients were included in the analysis and considered descriptively by characteristic (Table 4). Of the participants, 44.2% are women and 55.8% are men. No one classified their own gender as “diverse”. The average age is 71.1 years (SD = 14.8) with a range from 19 to 98 years. Most respondents (85.9%) were born in Germany. The median of education is a certificate of secondary education (29.2%), and 46.8% having completed a lower secondary school leaving certificate. 22.9% of participants have a high school diploma. The median of the highest professional qualification is “completed vocational training”, accounting for 71.5%. This is followed by a university degree at 13.8%. For this age cohort, the additional characteristics such as gender, migration status, and educational and occupational qualifications are representative of the population; there are only slight deviations (24–26).

**Table 4:** Characteristics of the participating patients.

Variable	*n* (%)	*M*	SD	Range
Sex/gender (as stated in the questionnaire)	388 (100)			
Female	171 (44.2)			
Male	217 (55.8)			
Age (years)	389 (100)	71.1	14.8	19–98
19–30	7 (1.8)			
31–40	13 (3.4)			
41–50	17 (4.3)			
51–60	43 (11.2)			
61–70	73 (18.8)			
71–80	111 (28.7)			
81–90	118 (30.4)			
91–98	6 (1.6)			
Country of birth	389 (100)			
Germany	334 (85.9)			
Other	55 (14.1)			
Graduation	390 (100)			
High school diploma	89 (22.9)			
Certificate of secondary				
education	114 (29.2)			
Lower secondary school				
leaving certificate	183 (46.8)			
No school leaving certificate	3 (0.8)			
Other	1 (0.2)			
Professional qualification	383 (100)			
Doctoral degree	8 (2.0)			
University degree	53 (13.8)			
Completed vocational training	274 (71.5)			
In vocational training	4 (1.1)			
No professional qualification	37 (9.5)			
Other	8 (2.0)			

*M* = mean; SD = standard deviation. The data is weighted according to care level and region.

The results regarding the need for and perception of GSC+ were measured using a set of six questions (Table 5). The first item shows that personal needs regarding nutrition selection are met (*M* = 3.03), and the second item shows that there was sufficient confidentiality during medical consultations (*M* = 3.09). Women are more satisfied than men, showing item 2 (*p* < 0.05). There is no average trend regarding information on gender-specific aspects in medical consultations. 45.1% would like more or rather more information, while 45.7% feel sufficiently, or rather sufficiently informed. 9.3% have no opinion. The age and gender of nurses were considered rather unimportant factors in patient care on average (items 4 and 5). Age was slightly more important to men (*p* < 0.05) and gender slightly more important to women (*p* < 0.01). Similarly, the gender of physicians was considered rather unimportant in patient care on average (item 6), with no significant difference between women and men. Overall, it appears that the need for GSC+ among patients is rather low or is already being met.

**Table 5:** Patients’ needs and perceptions regarding GSC+ by gender.

Item	*M* total	*M* women	*M* men	Difference (*M*_w_−*M*_m_)	*p*
1. Nutrition selection met personal needs	3.03	2.89	3.14	−0.25	0.085
2. Sufficient trust during medical consultations	3.09	3.27	2.95	0.32*	0.029
3. Adequate information on gender-specific aspects during medical consultations	2.60	2.56	2.62	−0.06	0.742
4. Age of nurse as a relevant factor	1.35	1.21	1.45	−0.24*	0.044
5. Same-sex nursing care is desired	1.64	1.96	1.39	0.58**	0.001
6. Same-sex medical care by a physician is desired	1.24	1.32	1.17	0.16	0.187

*M* = mean. Scale 1-4. The data is weighted according to care level and region.

* *p* < 0.05.

** *p* < 0.01.

*** *p* < 0.001.

The six items were summarized into a mean index (scale 1-4), because it summarizes the assessment of the GSC+ implementation measures in an index. Although the observed reliability is relatively low (Crohnbach´s alpha = 0.512), it may be regarded as acceptable in the context of an exploratory study. However, results involving this variable should be interpreted with appropriate caution; Factor analyses were not conducted.

The influence of sociodemographic characteristics on this index was calculated using multivariate linear regression with backward elimination (Table 6). The variables “country of birth” and “gender” identified the backward elimination as adequate independent variables. A regression with the inclusion method was calculated using these variables. The variables explain 5.0% of the variance in the need for GSC+ with a corrected *R*² of 0.045. Patients born in Germany have a significantly lower need for GSC+ on average than patients not born in Germany. In addition, women have a greater need for GSC+ than men.

**Table 6:** Regression analysis of patients’ needs/perceptions regarding GSC+.

Predictor	Coeff.	Beta	CI (95%)		*p*
Country of birth (1 = Germany)	−0.174**	−0.134	[−0.301;	−0.047]	0.008
Recorded sex/gender (1 = female)	0.164***	0.180	[0.074;	0.253]	<0.001
*R*^2^/Adj. *R*^2^	0.050/0.045				
*N*	383				

The data is unweighted.

* *p* < 0.05.

** *p* < 0.01.

*** *p* < 0.001.

Participants were also asked about their self-reported awareness of sex-related cardiovascular differences, specifically potential differences between women and men in blood pressure and myocardial infarction symptoms. Slightly more respondents (62%) were aware of the difference in myocardial infarction symptoms than in normal blood pressure values (51%). Women are more likely than men to be aware of sex-specific differences in both questions, but only significant for myocardial infarction symptoms. 47% of respondents had heard about GSC+ on television, radio, or read about it in magazines (Table 7).

**Table 7:** Patients’ self-reported awareness of GSC+ by sex.

Variable	*M* total	*M* women	*M* men	Difference (*M*_w_−*M*_m_)	*p*
Blood pressure	0.51	0.57	0.47	0.09	0.257
Myocardial infarction symptoms	0.62	0.72	0.54	0.18*	0.024
Information about GSC+ via media	0.47	0.54	0.42	0.12	0.152

*M* = mean. Scale 0-1. The data is weighted according to care level and region.

* *p* < 0.05.

** *p* < 0.01.

*** *p* < 0.001.

The two self-reported awareness items were combined into a mean index (scale 0–1). In the multivariate linear regression with backward elimination, country of birth, sex, school leaving certificate, and vocational qualification remained as independent variables. A regression was calculated using these variables with the inclusion method. The variables explained 8.4% of the variance in self-reported awareness of sex-related cardiovascular differences, with an adjusted *R*² of 0.072. On average, women reported greater awareness of sex-related cardiovascular differences than men. Patients with a high school diploma and a secondary school diploma reported greater awareness than patients with a lower secondary school leaving certificate or a lower qualification. Country of birth and having a university or doctoral degree had no significant influence. Overall, sociodemographic characteristics alone had relatively low explanatory power for self-reported awareness of sex-related cardiovascular differences. Higher self-reported awareness among women and patients with higher levels of school education was in line with expectations (Table 8).

**Table 8:** Regression analysis of patients’ self-reported awareness regarding GSC+.

Predictor	Coeff.	Beta	CI (95%)		*p*
Country of birth (1 = Germany)	0.100	0.085	[−0.018;	0.218]	0.096
Sex (1 = female)	0.160***	0.191	[0.077;	0.242]	0.000
High school diploma^a^	0.144*	0.153	[0.020;	0.267]	0.022
Certificate of secondary education^a^	0.106*	0.119	[0.010;	0.201]	0.030
University degree/doctoral degree^b^	0.112	0.106	[−0.016;	0.240]	0.085
*R*^2^/Adj. *R*^2^	0.084/0.072				
*N*	374				

The data is unweighted.

a Reference: lower secondary school leaving certificate, other, and no graduation.

b Reference: completed vocational training and no professional qualification.

* *p* < 0.05.

** *p* < 0.01.

*** *p* < 0.001.

### Comparison between groups on quantitative data

This section synthesizes findings across patients, nurses, and physicians to examine differences in the perceived implementation of GSC+ in inpatient cardiology. In addition, gender sensitivity is compared between nurses and physicians using the N-GAMS. The analysis provides insight into whether expectations, competence levels, and perceived implementation differ between nurses and physicians, and taking into account the patient's view.

### Gender sensitivity among healthcare professionals

Gender sensitivity among healthcare professionals was assessed using the N-GAMS. Overall, both physicians and nurses demonstrated moderate to high levels of gender sensitivity. However, physicians reported significantly higher overall gender sensitivity scores than nurses, with a mean score of 3.95 (SD = 0.73) compared to 3.46 (SD = 0.76) among nurses. This difference between professional groups was statistically significant (*p* < 0.001) (Table 9).

**Table 9:** Comparison of mean values for gender sensitivity.

Professional group	*M*	SD	*N*
Physicians	3.95	0.73	155
Nurses	3.46	0.76	109
Difference	−0.49***		
*p*-Value	<0.001		

Assumed equal variance. Data weighted by care level and region.

* *p* < 0.05.

** *p* < 0.01.

*** *p* < 0.001.

The comparison is based on aggregated scale scores and indicates a systematic difference in overall gender sensitivity between the two professional groups. Contextual insights from the profession-specific quantitative analyses show that, among physicians, high gender sensitivity is primarily reflected in strong agreement with items emphasizing the clinical relevance of sex- and gender-related differences for diagnostic accuracy and treatment quality (19). Among nurses, gender sensitivity is likewise present but more heterogeneous across items, with lower and more variable agreement particularly in areas related to the practical integration of gender-sensitive considerations into routine care processes.

Taken together, these findings suggest that the observed group difference reflects variation in professional orientation and perceived scope of action in relation to GSC+.

### Perceived implementation of GSC+ across groups

Across all questionnaires, the final question was: “To what extent do you think gender-sensitive care is implemented on this ward?” (answer code 1–4). The mean values were compared across the groups. Significant differences were observed in the perceived implementation of GSC+ across stakeholder groups. Overall, patients reported the highest levels of perceived implementation, followed by nurses, whereas physicians consistently rated the degree of implementation as lowest. The overall group difference was statistically significant (*p* < 0.001), indicating systematic variation in how GSC+ is assessed across patients and professional groups. Patients' ratings suggest a comparatively positive evaluation of GSC+ in everyday hospital practice. Nurses reported moderately lower implementation levels, while physicians expressed the most critical assessments (Table 10).

**Table 10:** ANOVA for comparing the mean values of the assessment of the implementation of GSC+.

Professional group	*M*	SD	*N*
Physicians	2.45	0.59	138
Nurses	2.91	0.81	91
Patients	3.04	0.65	336
*p*-Value	<0.001		

The data is weighted according to care level and region.

* *p* < 0.05.

** *p* < 0.01.

*** *p* < 0.001.

Post-hoc analyses using Tukey's HSD test were conducted to further examine pairwise differences between stakeholder groups. These analyses confirmed that physicians' assessments of the implementation of GSC+ differed significantly from those of both nurses and patients. Physicians rated the level of implementation significantly lower than nurses [mean difference = 0.46, 95% CI (0.25; 0.67), *p* < 0.001] and significantly lower than patients [mean difference = 0.59, 95% CI (0.43; 0.75), *p* < 0.001].

In contrast, no statistically significant difference was observed between nurses' and patients' assessments of implementation [mean difference = 0.13, 95% CI (−0.05; 0.31), *p* = 0.222]. This finding indicates a high degree of concordance between nurses and patients with regard to their overall evaluation of how GSC+ is implemented in everyday clinical practice (Table 11).

**Table 11:** Turkey HSD *post-hoc* text on the mean comparison of the implementation of GSC+.

Group	Difference	CI (95%)		*p*-Value
Physicians vs nurses	0.46***	[0.25;	0.67]	<0.001
Physicians vs patients	0.59***	[0.43;	0.75]	<0.001
Nurses vs patients	0.13	[−0.05;	0.31]	0.222

The data is weighted according to supply level and region.

* *p* < 0.05.

** *p* < 0.01.

*** *p* < 0.001.

### Cross-group synthesis of perspectives

Taken together, the group comparisons reveal a differentiated picture of GSC+ in inpatient cardiology. Across professional groups, physicians report higher overall gender sensitivity than nurses, as measured by the N-GAMS. At the same time, physicians rate the implementation of GSC+ in everyday clinical practice lower than both nurses and patients. It is notable that the group with the highest reported gender sensitivity simultaneously reports the lowest perceived level of implementation.

Patients, in contrast, report the highest overall assessments of GSC+ implementation. Nurses' assessments are located between those of physicians and patients, both with regard to gender sensitivity and perceived implementation.

The data reflects systematic differences in reported gender sensitivity and perceived implementation levels. While gender sensitivity and perceived implementation do not align uniformly across stakeholder groups, the findings consistently show that higher reported gender sensitivity is not necessarily accompanied by higher perceived implementation of GSC+.

The following Figure 2 shows an overview of the study results.

**Figure 2:**
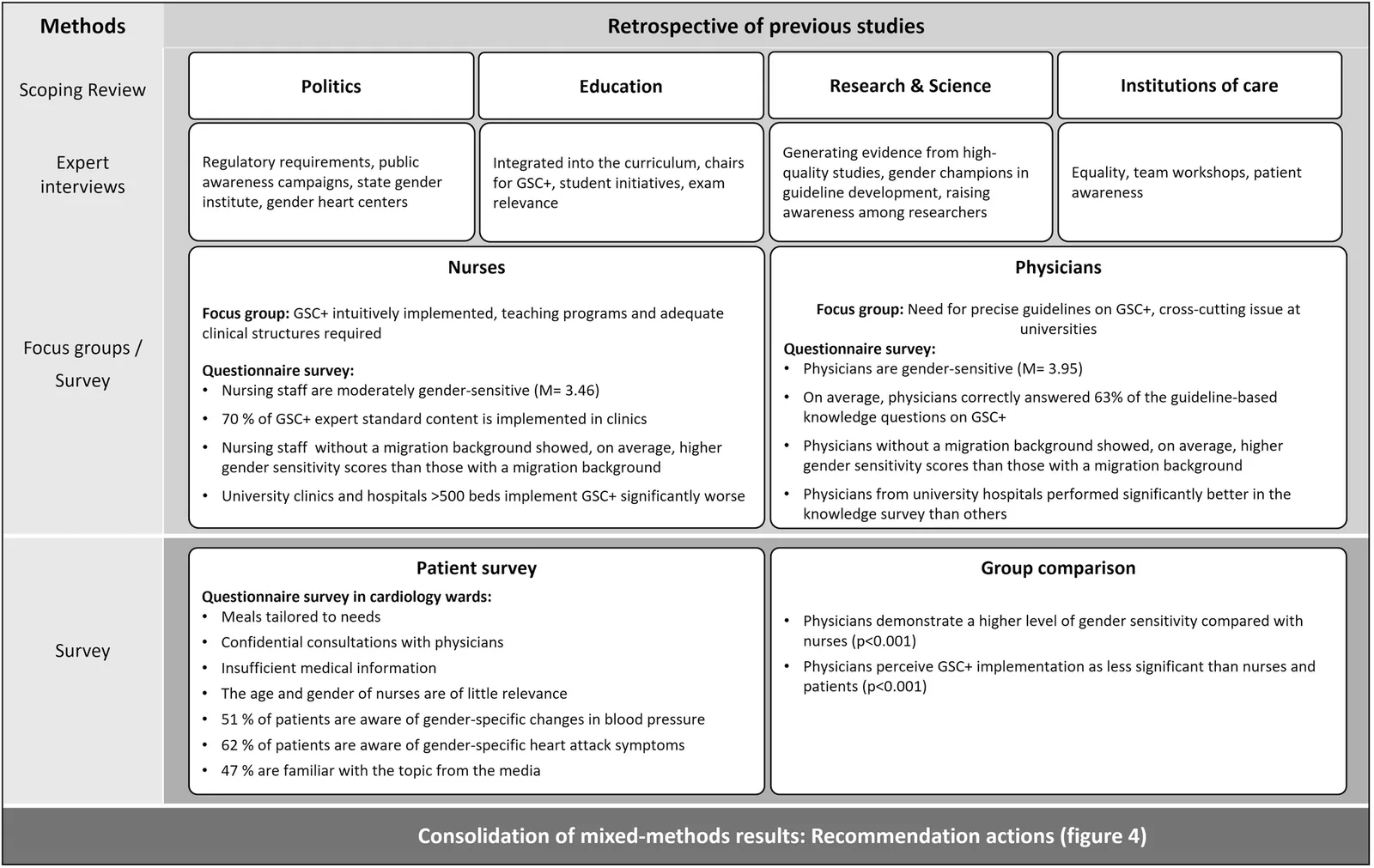
Overview of the study results.

The following section addresses the predefined hypotheses derived from the study protocol (12) and evaluates them based on the triangulated findings of the scoping review, the qualitative focus groups with physicians and nurses, and the quantitative survey among healthcare professionals and patients.

Hypothesis 1: Despite the availability of gender-sensitive recommendations in clinical guidelines, their implementation in routine practice remains limited due to insufficient knowledge.

The findings partially support this hypothesis. Across professional groups, moderate to high levels of gender sensitivity were observed (physicians: *M* = 3.95, SD = 0.73; nurses: *M* = 3.46, SD = 0.76). However, the perceived implementation of GSC+ in routine practice remained limited, particularly from the physicians' perspective (physicians: *M* = 2.45, SD = 0.59; nurses: *M* = 2.91, SD = 0.81). Qualitative findings and results from the scoping review further indicate that gender-specific content in guidelines and nursing expert standards is often insufficiently concrete or operationalized. This suggests that the limited implementation is not solely attributable to a lack of knowledge but rather reflects a gap between abstract knowledge and its practical application.

Hypothesis 2: Working conditions, particularly staff shortages and economic constraints, are key factors contributing to the low level of implementation of GSC+.

This hypothesis is supported. Qualitative findings from physicians and nurses consistently identified time pressure, staff shortages, and high workload as major barriers to implementing GSC+. These barriers are also reflected in the scoping review, which highlights organizational and structural constraints as central impediments (15). Nurses in particular emphasized that such conditions restrict the systematic consideration of gender- and diversity-related needs despite individual awareness (9).

Hypothesis 3: The requirements for gender-specific differentiation as formulated in clinical guidelines are insufficiently known.

Partial support was found for this hypothesis. While clinical guidelines were described as central reference tools, both qualitative findings and the scoping review suggest that gender-specific recommendations are often fragmented, insufficiently visible, or not sufficiently operationalized for routine practice. Quantitative data further indicate that physicians correctly answered, on average, 63% of the knowledge-based questions on gender-specific aspects of cardiovascular guidelines, pointing to a moderate but incomplete level of knowledge. Patient survey data likewise revealed heterogeneous patterns of self-reported awareness, with higher awareness among women and patients with higher educational levels.

Hypothesis 4: The requirements for gender-specific differentiation as formulated in clinical guidelines are not fully compatible with everyday clinical practice.

This hypothesis is supported by findings across all methodological components. Physicians and nurses reported that gender-sensitive differentiation is rarely embedded in workflows, documentation systems, or standard operating procedures and is often perceived as an additional rather than integral component of care. The scoping review similarly highlights limited workflow compatibility and lack of structural integration as key implementation barriers (15).

Hypothesis 5: The requirements for gender-specific differentiation do not fully correspond to the attitudes of healthcare professionals.

This hypothesis is partially supported. Overall attitudes toward GSC+ were positive across professional groups. However, higher levels of gender sensitivity (physicians: *M* = 3.95, SD = 0.73; nurses: *M* = 3.46, SD = 0.76) did not translate into higher perceived implementation of gender-sensitive care (physicians: *M* = 2.45, SD = 0.59; nurses: *M* = 2.91, SD = 0.81). This finding suggests that structural, organizational, and systemic factors play a more decisive role than individual attitudes alone.

Overall, the hypothesis assessment indicates that insufficient evidence, structural constraints, limited operationalization of gender-sensitive requirements, and insufficient institutional embedding were consistently identified in relation to implementation gaps in GSC+ rather than by negative attitudes or a lack of awareness among healthcare professionals.

## Discussion

This exploratory mixed-methods study HeartGap examined the current status of GSC+ in inpatient cardiology by integrating the perspectives of patients, physicians, and nurses. By combining quantitative group comparisons with qualitative insights and contextual evidence, the findings reveal a differentiated picture of how GSC+ is understood, evaluated, and enacted in inpatient cardiology practice by professionals and perceived by patients. The overarching aim of the study is to identify the status quo of EbM and evidence-based nursing (EBN) regarding guidelines and expert standards, investigate their implementation in healthcare practice, and derive recommendations for action, considering the quintuple aim to enhance the healthcare system in quality (Figure 3).

**Figure 3:**
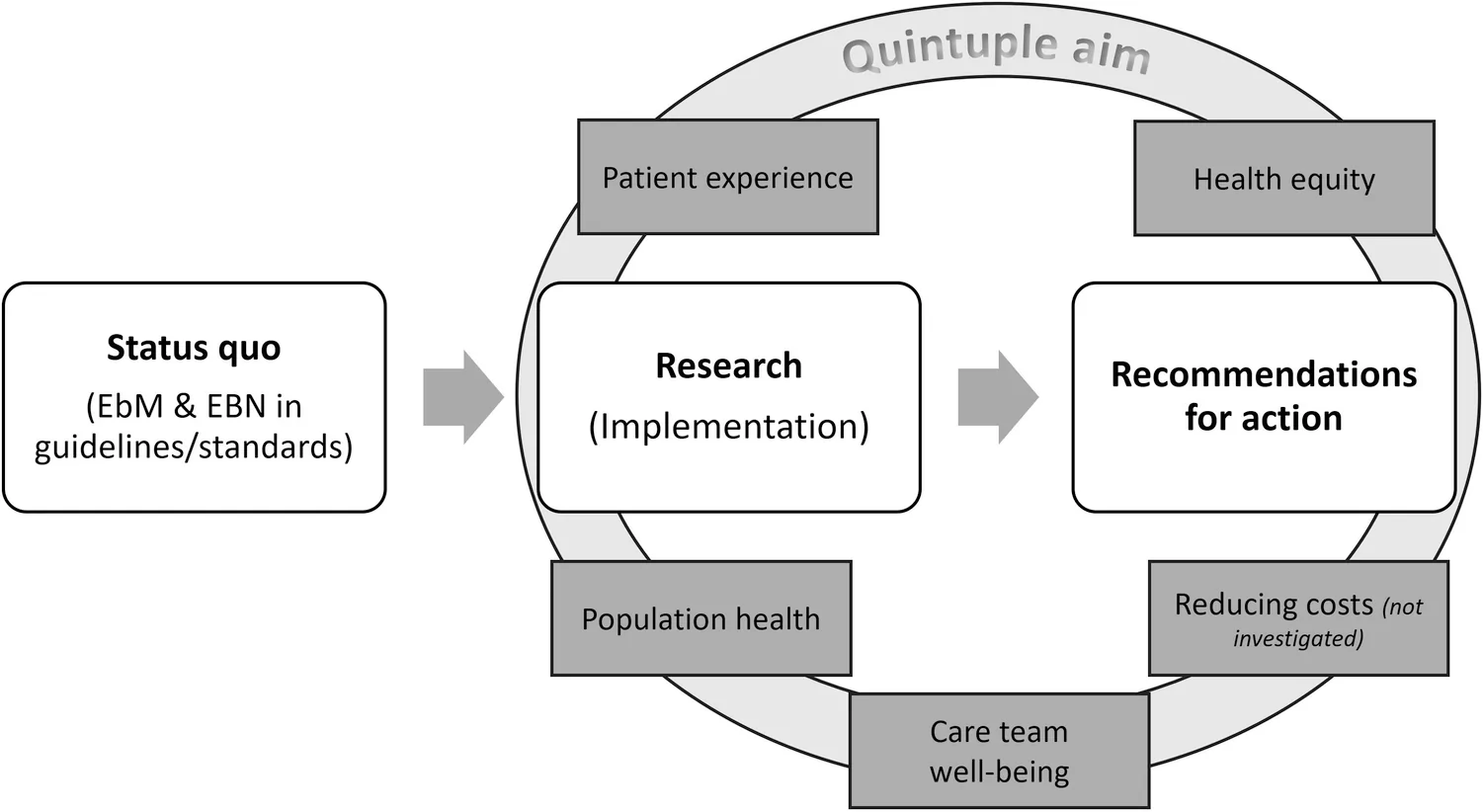
Mixed-methods research and recommendation derivation under consideration of the quintuple aim.

Rather than indicating a uniform level of implementation, the results point to systematic differences shaped by professional roles, evaluative reference frames, and organizational conditions.

A central finding of the study is the marked discrepancy between reported gender sensitivity and perceived implementation across professional groups. Physicians reported the highest levels of gender sensitivity, yet simultaneously assessed the implementation of GSC+ significantly lower than both nurses and patients. Nurses, in contrast, reported slightly lower gender sensitivity while perceiving higher levels of implementation. This constellation suggests that higher gender sensitivity does not automatically translate into higher perceived implementation.

Patients reported the highest levels of perceived implementation of GSC+ while expressing comparatively low preferences for several additional GSC+ measures assessed in the survey. The comparatively low preference for same-sex care should not be interpreted as indicating a generally low need for GSC+, as provider–patient gender concordance represents only one specific dimension of GSC+. GSC+ encompasses a broader range of sex- and gender-related considerations in diagnostics, treatment, communication, interaction, and organizational care. The patient survey revealed differences in self-reported awareness of sex-related cardiovascular aspects, particularly by sex and educational level. This constellation raises the hypothesis of an “expectation paradox”: positive patient evaluations may coexist with incomplete implementation of GSC+. This interpretation should be considered hypothesis-generating rather than an empirically tested mechanism. One possible explanation is that patients primarily evaluate visible and experiential aspects of care, such as trust, communication quality, perceived attentiveness, and respect, whereas biomedical, diagnostic, therapeutic, or structural components of GSC+ may be less directly observable to them. Furthermore comparatively positive patient evaluations may be influenced by limited awareness of what evidence-based GSC+ entails, social desirability or acquiescence effects in the inpatient setting, and researcher-assisted questionnaire completion. In addition, differences in item wording and response scales across stakeholder groups may have contributed to differences in reported implementation and should be considered when interpreting cross-group comparisons. The findings should also be interpreted in light of the specific healthcare context,s the study exclusively included patients from inpatient cardiology. In clinical areas characterized, e.g., by more body-related, intimate, or long-term care relationships, such as gynecology, urology, psychiatry, other dimensions of GSC+, including same-sex care, intimacy, or experiences of shame, may be perceived as more relevant and evaluated differently. In outpatient settings, where care relationships are often longer-term and embedded in patients' everyday lives, expectations and evaluations of GSC+ also may differ from those observed in inpatient acute care. Perceived patientśneeds and evaluative criteria for GSC+ vary across clinical specialties and care settings.

Physicians’ more critical assessments may be interpreted as an expression of a stronger orientation toward formal guidelines, evidence-based recommendations, and standardized documentation. Greater awareness of sex- and gender-related evidence may therefore increase sensitivity to gaps, inconsistencies, and missing structural anchors in routine care. In this sense, higher gender sensitivity may lead to more critical judgments, as problems become more visible (27).

Nursing professionals, in comparison, appear to evaluate implementation primarily through the lens of everyday practice. Their understanding of GSC+ is more strongly action- and relationship-oriented, emphasizing communication, situational responsiveness, attention to privacy, and individualized interaction with patients (9). Consequently, implementation is experienced as more present in daily care, even when formal standards, documentation, or explicitly gender-sensitive protocols are no priority and GSNC+ is more informal. These differences highlight the role of professional socialization and perceived scopes of action in shaping how GSC+ is conceptualized and assessed.

Beyond differing evaluative lenses, the findings also point to professional-specific understandings of responsibility for GSC+. While physicians tend to locate responsibility within a rather formal decision-making structure and guideline adherence, nurses more often perceive GSC+ as embedded in relational and situational care practices. In the absence of clearly defined institutional responsibilities and shared interprofessional standards, this divergence risks producing diffuse accountability, in which GSC+ is implicitly expected but not explicitly assigned.

The findings underline that GSC+ is neither a uniformly defined nor consistently implemented construct, but one that is perceived, operationalized, and evaluated differently by patients, physicians, and nurses, depending on professional roles, experiential reference frames, personal preference, and everyday practice contexts. These differentiated perceptions do not indicate that GSC+ is optional or purely preference-based. Rather, they underscore the need for a clearly defined minimum standard of GSC+ that ensures diagnostic accuracy, treatment quality, equity, and patient safety, while allowing flexibility in how additional needs are addressed across individual patients and professional contexts (28).

Many gender-sensitive measures, especially those related to biomedical differentiation in diagnostics or treatment decisions, remain largely invisible to patients due to differences in medical knowledge between patients and healthcare professionals. As a result, healthcare may be experienced as individualized and responsive even when gender-sensitive differentiation is not systematically embedded or explicitly communicated. Implicit norms of equal treatment may further reinforce this pattern. Equal treatment is often perceived as fair, even when relevant differences related to sex, gender, or social position are not actively addressed. This finding opens a broader discussion about equality versus equity in healthcare and highlights the challenge of making differentiated care both visible and meaningful from the patient perspective.

Furthermore, differences in perceived needs among women and patients with a migration background underline the relevance of socialization and social positioning in shaping expectations, needs, health literacy, and experiences of care. Women reported both higher awareness of sex-related cardiovascular differences and greater needs for GSC+ which may reflect increased societal visibility and public discourse around gender and health. Patients with a migration background likewise reported higher needs for GSC+, pointing to intersecting dimensions of gender, culture, language, and access to care (29). Although the explanatory power of sociodemographic variables was modest, these findings underline the importance of intersectional perspectives that remain insufficiently integrated into current care structures. At present, GSC+ is still predominantly framed in binary gender terms (30, 31), while broader intersections with migration, socioeconomic position, education, and age are only partially addressed in healthcare structures (32).

The limited integration of intersectional dimensions should not be understood solely as an empirical gap, but rather as a reflection of prevailing healthcare models that continue to prioritize standardized categories over complex social positioning. Advancing GSC+ therefore requires not only greater awareness of intersectionality but also structural adaptations that enable healthcare systems to respond to overlapping and cumulative dimensions of vulnerability.

Across healthcare professionals, GSC+ was frequently perceived more as an additional rather than integral component of care. Both physicians and nurses described gender-sensitive differentiation as insufficiently operationalized within clinical guidelines, nursing expert standards, documentation systems, and quality management instruments. As a result, implementation of GSC+ remains highly dependent on individual engagement and situational discretion. Under conditions of time pressure, staff shortages, and high workload, such additive requirements are particularly difficult to realize, which helps explain why positive attitudes and awareness do not translate into consistent implementation (9, 33).

The organizational context therefore emerges as an important factor associated with the implementation of GSC+. Individual knowledge and motivation alone appear insufficient to explain perceived implementation. Across the study components, organizational structures, leadership priorities, and institutional accountability were consistently identified as relevant factors shaping how care principles are translated into everyday practice. Nursing professionals occupy a key position in operationalizing people-centered and GSC+ due to their continuous patient contact.

Leadership and organizational culture play a central yet underdeveloped role in this context. Leadership commitment influences priority setting, resource allocation, and the normalization of gender-sensitive practices within clinical routines. Without active leadership engagement, GSC+ risks remaining an individual concern rather than an organizational responsibility (34, 35).

Within the broader concept of people-centered care, the findings demonstrate that people-centeredness does not automatically result solely from healthcare professionals' positive attitudes. It requires concrete operationalization within clinical routines and organizational systems. GSC+ can be understood as a practical expression of people-centered care that translates abstract principles into clinically relevant action (8). Furthermore, GSC+ should not be reduced to a political or ideological debate. Although discussions of gender are frequently politicized, it is essential to distinguish these debates from medical and healthcare-related considerations, where gender has direct and evidence-based implications for diagnostic accuracy, treatment quality, equity, and patient safety.

In cardiology, where sex- and gender-related differences affect symptom recognition, diagnostic pathways, therapeutic decisions, and outcomes, insufficient implementation of GSC+ may have consequences that extend beyond questions of fairness to directly impact care quality and patient safety (36). GSC+ is therefore not an optional or supplementary consideration, but indispensable for high-quality, people-centered, and safe care. Strengthening the structural integration of GSC+ within guidelines, education, organizational routines, and quality assurance frameworks represents a key leverage point for closing persistent gender health gaps and advancing people-centered healthcare (15, 37). In the long term, GSC+ is intended to support the Quintuple aims and improve patient experiences, population health, care team well-being, cost reduction, and health equity.

## Limitation

The patient survey was conducted with the option of an accompanied survey. This means that the researchers read the questions and possible answers aloud to the inpatients. Given the situation the patients were in, most of them utilize this option. The inpatients had suffered a cardiac event, such as a heart attack or heart failure, and were in the hospital. It should be noted that, despite the claim to objectivity, patient surveys may have been accompanied by distortions. A further limitation concerns the operationalization of sex and gender. The patient questionnaire used a single German-language item (“Geschlecht”) and did not separately assess sex assigned at birth and current gender identity. Therefore, the dataset does not allow sex-related and gender-related associations to be disentangled, and the quantitative comparisons are restricted to women and men. The findings from these analyses should consequently not be generalized to gender-diverse populations. Future studies should assess sex assigned at birth and gender identity separately and include response options that adequately capture gender-diverse identities. Furthermore, accompanied questioning tends to encourage socially desirable responses. In particular, the two self-reported awareness items could have been affected by social desirability bias, as participants may have been reluctant to report that they were unfamiliar with the respective sex-related cardiovascular differences. Moreover, these items assessed respondents’ perceived awareness rather than objectively testing their knowledge of specific symptoms, blood pressure trajectories, or diagnostic thresholds. The findings should therefore not be interpreted as measures of objective cardiovascular knowledge.

The survey period for the cross-sectional study was extended by a few months due to recruitment difficulties. Nevertheless, the successive survey may have been influenced by changes in external factors, for example, more public relations work on GSC+.

The study has considered four of the quintuple aims in particular. Requirements for patient experience, health equity, population health, and care team well-being in context of GSC+ were identified. The aim of “reducing costs” was not explicitly investigated.

## Conclusion

The consideration of sex and gender in patient care constitutes a key component of high-quality, people-centered healthcare. This exploratory mixed-methods study demonstrates that, despite growing awareness of GSC+, substantial gaps remain between available evidence, professional attitudes, and structural implementation in inpatient cardiology. By integrating the perspectives of patients, physicians, and nurses, the study highlights that GSC+ is perceived and enacted differently across stakeholder groups and remains insufficiently embedded in routine care processes.

The findings indicate that patients generally evaluate the implementation of GSC+ positively despite variation in self-reported awareness of sex-related cardiovascular differences, particularly among men and patients with lower educational levels.. Healthcare professionals, in contrast, demonstrate moderate to high gender sensitivity and guideline orientation, yet consistently report limited implementation of gender-sensitive differentiation in everyday practice. These discrepancies underscore that awareness and positive attitudes alone are insufficient to ensure systematic implementation. Instead, GSC+ requires structural anchoring, organizational support, and coordinated action across multiple system levels.

Based on the integrated results of the HeartGap project and supported by an interdisciplinary expert advisory board, the following recommendations for action were derived (Figure 4):

**Figure 4:**
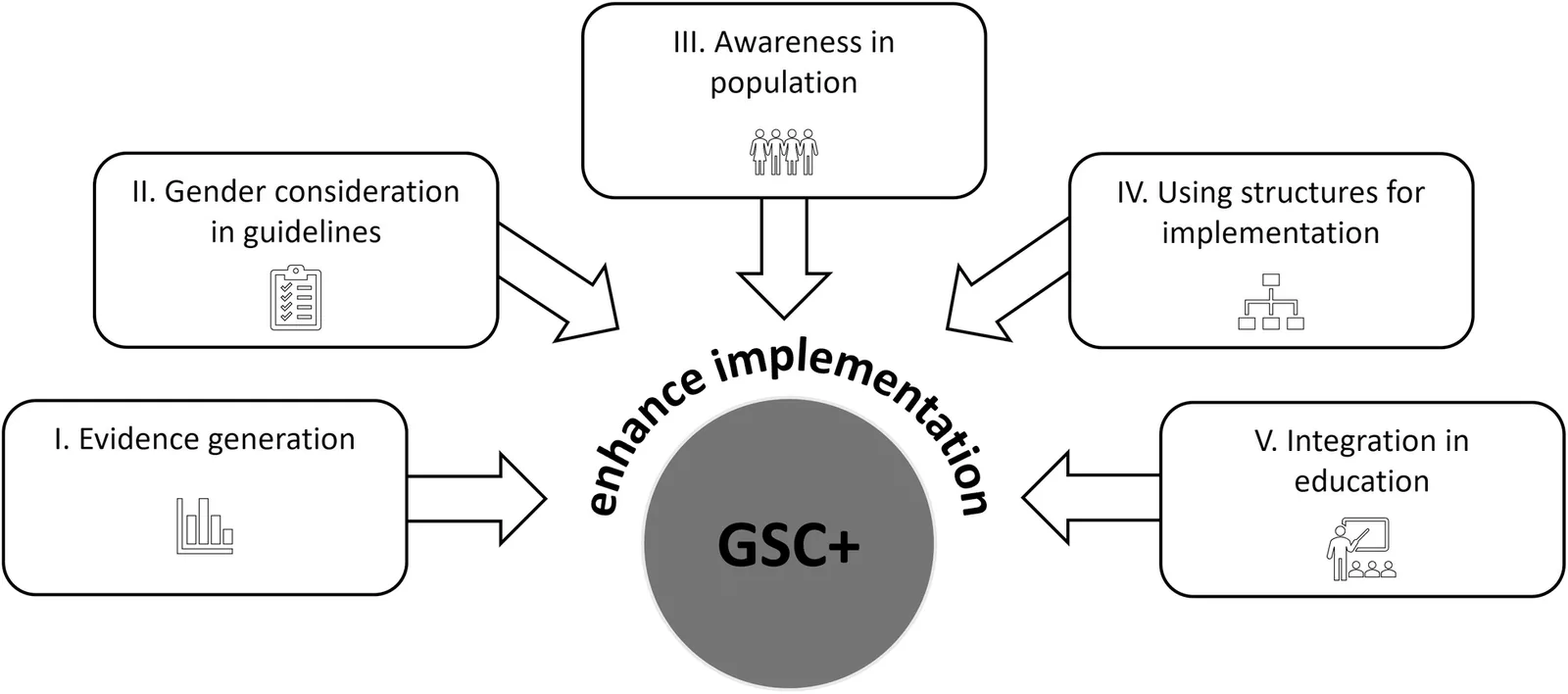
Recommendations for action.

### Recommendation I: strengthen the evidence base for GSC+

Further high-quality studies are needed to systematically generate and consolidate evidence on sex- and gender-specific differences in diagnostics, treatment, and care processes. Robust evidence is essential to inform clinical guidelines, support decision-making, and legitimize GSC+ as a quality and safety standard within healthcare systems.

In line with our findings and previous research, current evidence remains fragmented, and women and gender-diverse populations are still underrepresented in clinical studies. This limits not only the generalizability of findings but also the development of precise, evidence-based recommendations, thereby contributing to uncertainty in clinical decision-making and inconsistent implementation.

### Recommendation II: systematically integrate gender sensitivity into clinical guidelines and expert standards

Gender-sensitive recommendations should be made more visible, concrete, and operational within medical guidelines and nursing expert standards. Clear guidance on when and how gender-specific differentiation is required can facilitate translation into routine practice and reduce reliance on individual discretion. Our results indicate that existing recommendations are often perceived as insufficiently specific, fragmented, or difficult to operationalize. As a result, clinicians may lack clear decision-support in everyday practice, which contributes to variability in implementation and reinforces the gap between guideline content and clinical application.

### Recommendation III: raise awareness and expand knowledge among both the population and healthcare professionals

Targeted information and communication strategies are needed to address persistent knowledge gaps among patients and healthcare professionals. Improving public and professional understanding of gender-specific health aspects can support informed decision-making, enhance patient engagement, and increase demand for GSC+. Consistent with our findings, knowledge levels were moderate but unevenly distributed across both professionals and patients. This suggests that awareness alone is insufficient and needs to be complemented by structured, context-specific knowledge that supports clinical reasoning and patient–provider communication.

### Recommendation IV: utilize organizational structures to support implementation of GSC+

Healthcare institutions should embed GSC+ within organizational routines, documentation systems, quality management instruments, and leadership responsibilities. Structural integration is a prerequisite for sustainable implementation, particularly under conditions of time pressure, staff shortages, and high workload. Qualitative findings and stakeholder perspectives highlighted that structural barriers, such as limited time, competing priorities, and insufficient integration into workflows, currently restrict the consistent application of GSC+ despite individual awareness. Without embedding GSC+ into routine processes, implementation remains dependent on individual initiative and is therefore unlikely to be sustained.

### Recommendation V: integrate GSC+ into education and training across professions

Gender-sensitive and intersectional perspectives should be systematically incorporated into undergraduate education, postgraduate training, and continuing professional development for all healthcare professions. Education plays a central role in shaping professional socialization and fostering a shared understanding of GSC+ as an integral component of high-quality practice. The observed variability in knowledge and implementation underscores the need for structured and longitudinal integration of GSC+ into medical and nursing education. Early and continuous exposure is essential to ensure that GSC+ becomes a routine component of clinical reasoning rather than an additional or optional consideration.

In conclusion, GSC+ should not be regarded as an optional or supplementary aspect of healthcare, but as a core element of quality, equity, and patient safety. Strengthening its structural integration across research, education, policy, and healthcare organizations represents a critical leverage point for closing persistent gender health gaps and advancing people-centered healthcare.

## Data Availability

The raw data supporting the conclusions of this article will be made available by the authors, without undue reservation.
