# Supplementary material for: Gender sensitivity and its implementation in inpatient cardiology: a mixed-methods study of patient and healthcare professional perspectives
Source: Front Glob Womens Health. 2026 Oct 16;7:1871546. doi: 10.3389/fgwh.2026.1871546 (PMC13719549; doi:10.3389/fgwh.2026.1871546)
Supplement: Supplementary file 1 [file Datasheet1.pdf]

HeartGap Questionnaire for Patients
(English Version)

Dear Participant,

Thank you very much for your willingness to participate in this survey.

As part of the Innovation Fund–funded project *HeartGap*, we would like to ask about your experiences and assessments during your recent hospital stay. The Hannover Medical School (MHH) and the Private Research Institute for Health and System Design (figus GmbH) have been commissioned by the Federal Joint Committee to conduct the evaluation.

The survey takes approximately 10 minutes. All responses will be analyzed anonymously, and no conclusions can be drawn about your identity. The results will be used to assess the current state of gender-sensitive care. Data will not be shared with third parties. Anonymized data may be published in scientific journals.

Please complete the questionnaire as fully as possible and answer based on your first impression.

Thank you for your participation!

Questions?
Judith Mollenhauer, M.Sc. (Research Associate, figus Institute)
Email: j.mollenhauer@figus.koeln
Phone: +49 221 29257382

Sophia Sgraja, M.A. (Research Associate, Hannover Medical School)
Email: sgraja.sophia@mh-hannover.de
Phone: +49 511 5325417

Data Protection Statement

Participation in this survey is voluntary. No names or contact details will be collected. However, demographic data (e.g., age, gender, education) will be requested.

Before starting the survey, you will receive detailed information about your rights and will be asked for your consent.

- Participation is voluntary and can be withdrawn at any time.
- Your participation is anonymous; responses cannot be traced back to you.
- Due to anonymization, deletion of individual responses is not possible after submission.
- Data will be stored for at least 10 years and then deleted.

Please tick below:
☐ I confirm that I have read the information above and agree to participate in this survey.

Part A: About the Person

A1. Gender:

- Female
- Male
- Diverse

A2. Age (in years):
[Free text]

A3. Born in Germany?

- Yes
- No

A4. Highest school qualification:

- (Technical) high school diploma / university entrance qualification
- Secondary school certificate
- Lower secondary school certificate
- No school qualification
- Other: [Free text]

A5. Highest professional qualification:

- Habilitation
- Doctorate
- University degree (Bachelor, Master, etc.)
- Completed vocational training/apprenticeship
- No professional qualification
- Currently in education / training
- Other: [Free text]

Part B: Your Hospital Stay

B1. Please indicate to what extent you agree with the following statements.
(Scale: Strongly agree – Agree – Disagree – Strongly disagree – Don't know)

- The hospital catering met your personal needs (e.g., health-related, religious, cultural).
- There was sufficient time and privacy during medical consultations to discuss your illness and life circumstances.

- In your opinion, should more attention be paid during medical consultations to gender-specific aspects (e.g., medication dosage, side effects)?

B2.
(Scale: Strongly agree – Agree – Disagree – Strongly disagree – Don’t know)

- Is the age of the nursing staff an important factor for you to feel comfortable during care (e.g., washing, dressing)?

B3.
(Scale: Strongly agree – Agree – Disagree – Strongly disagree – Don’t know)

- As a woman, do you feel more comfortable being cared for by a female nurse / as a man by a male nurse?

B4. With regard to the previous question:
If applicable, was your preference taken into account?

- Yes
- No
- Not applicable

B5.
(Scale: Strongly agree – Agree – Disagree – Strongly disagree – Don’t know)

- As a woman, do you feel more comfortable being treated by a female physician / as a man by a male physician?

B6. With regard to the previous question:
If applicable, was your preference taken into account?

- Yes
- No
- Not applicable

Part C: Questions on Medicine

C1. Did you know that normal blood pressure differs between women and men?

- Yes
- No

C2. Did you know that symptoms of a heart attack can differ between men and women?

- Yes
- No

C3. Have you ever heard or read about gender medicine or gender-sensitive care (e.g., differences in symptoms or treatment between men and women)?

- Yes
- No

C4. Final Question:
(Scale: Very well – Well – Poorly – Not at all – Cannot judge)

To what extent is gender-sensitive care implemented on this ward (e.g., respect for privacy, religious needs such as space for prayer, consideration of migration background such as language barriers or dietary habits)?

C5. Additional comments (optional):
[Free text]

Thank you very much for your participation!

If you have further questions, please contact:
j.mollenhauer@figus.koeln

or
sgraja.sophia@mh-hannover.de
