# Supplementary material for: Gender sensitivity and its implementation in inpatient cardiology: a mixed-methods study of patient and healthcare professional perspectives
Source: Front Glob Womens Health. 2026 Oct 16;7:1871546. doi: 10.3389/fgwh.2026.1871546 (PMC13719549; doi:10.3389/fgwh.2026.1871546)
Supplement: Supplementary file 2 [file Datasheet2.pdf]

Appendix 1: GRAMMS reporting criteria

**GRAMMS Criterion**	**Focus Area**	**Transfer in Present Study**
Justification for mixed methods	State the justification for utilising mixed methods in this study	The study´s overarching aim was to develop recommendations for action. To fully analyze this complex and multifaceted context, it was necessary to utilize a combination of methods.
Description of design	Describe the type, priority and sequence of the different methods of study employed	Sequential mixed-methods design<br>Scoping Review needed to develop the interview guide for focus group interviews. The interviews and questionnaire surveys were conducted sequentially.<br>The results from the scoping review and the qualitative and quantitative studies inform the derivation of recommendations for action.
Methods detail	Describe the sampling approach for both quantitative and qualitative data collection and description of data analysis method for both methods	Qualitative studies: The recruitment was conducted through the selected pilot hospitals. The data were content analyzed.<br>Quantitative studies (health care professionals): The identified adequate hospitals (meeting the inclusion criteria) were alerted to the questionnaire survey through a process involving multiple reminders. The data were statistically analyzed (descriptive and inferential).<br>Quantitative study (patients): Patients were recruited from the wards of selected, suitable hospitals. The data were statistically analyzed (descriptive and inferential).
Integration	State how the quantitative and qualitative data was integrated	Data integration made it possible to interpret the results. A content integration was carried out to derive recommendations for action.

Method limitations	Identify the limitations associated with combining the two methods used for the study (mixed methods)	The qualitative and quantitative studies were conducted concurrently but at different times and were combined in content to derive recommendations for action. No further methodological limitations were identified.
Insights from integration	Provide details of any new knowledge gained through the integration of quantitative and qualitative data	-Measures to increase gender sensitivity<br>-Measures to develop guidelines and expert standards with regard to gender sensitivity<br>-Comparison of patients´perceptions versus those of health care professionals<br>-The expert advisory board confirmed the recommendations for action derived in advance from mixed-methods approach and specified them from a practical perspective
